# Metabolomic Analysis Reveals Metabolic Disturbance in the Cortex and Hippocampus of Subchronic MK-801 Treated Rats

**DOI:** 10.1371/journal.pone.0060598

**Published:** 2013-04-05

**Authors:** Liya Sun, Juan Li, Kejun Zhou, Ming Zhang, Jinglei Yang, Yang Li, Baohu Ji, Zhao Zhang, Hui Zhu, Lun Yang, Guang He, Linghan Gao, Zhiyun Wei, Kejian Wang, Xue Han, Weiqing Liu, Liwen Tan, Yihua Yu, Lin He, Chunling Wan

**Affiliations:** 1 Bio-X Institutes, Key Laboratory for the Genetics of Developmental and Neuropsychiatric Disorders, Ministry of Education, Shanghai Jiao Tong University, Shanghai, People’s Republic of China; 2 Institutes for Nutritional Sciences, Shanghai Institute of Biological Sciences, Chinese Academy of Sciences, Shanghai, People’s Republic of China; 3 East China Normal University, Department of Physics, Shanghai, People’s Republic of China; 4 Second Xiangya Hospital, Central South University, Institute of Mental Health, Changsha, People’s Republic of China; 5 CSIRO Animal, Food and Health Sciences, Queensland Bioscience Precinct, St. Lucia, Brisbane, Queensland, Australia; Torrey Pines Institute for Molecular Studies, United States of America

## Abstract

**Background:**

Although a number of proteins and genes relevant to schizophrenia have been identified in recent years, few are known about the exact metabolic pathway involved in this disease. Our previous proteomic study has revealed the energy metabolism abnormality in subchronic MK-801 treated rat, a well-established animal model for schizophrenia. This prompted us to further investigate metabolite levels in the same rat model to better delineate the metabolism dysfunctions and provide insights into the pathology of schizophrenia.

**Methods:**

Metabolomics, a high-throughput investigatory strategy developed in recent years, can offer comprehensive metabolite-level insights that complement protein and genetic findings. In this study, we employed a nondestructive metabolomic approach (1H-MAS-NMR) to investigate the metabolic traits in cortex and hippocampus of MK-801 treated rats. Multivariate statistics and ingenuity pathways analyses (IPA) were applied in data processing. The result was further integrated with our previous proteomic findings by IPA analysis to obtain a systematic view on our observations.

**Results:**

Clear distinctions between the MK-801 treated group and the control group in both cortex and hippocampus were found by OPLS-DA models (with R^2^X = 0.441, Q^2^Y = 0.413 and R^2^X = 0.698, Q^2^Y = 0.677, respectively). The change of a series of metabolites accounted for the separation, such as glutamate, glutamine, citrate and succinate. Most of these metabolites fell in a pathway characterized by down-regulated glutamate synthesis and disturbed Krebs cycle. IPA analysis further confirmed the involvement of energy metabolism abnormality induced by MK-801 treatment.

**Conclusions:**

Our metabolomics findings reveal systematic changes in pathways of glutamate metabolism and Krebs cycle in the MK-801 treated rats’ cortex and hippocampus, which confirmed and improved our previous proteomic observation and served as a valuable reference to the etiology research of schizophrenia.

## Introduction

Schizophrenia is a severe and complicated mental disorder that seriously impairs human independence and imposes a significant burden on society [1]. Both hereditary and environmental factors contribute to this disease. Much has been done in the past decades to unravel the pathogenesis of schizophrenia, leading to various hypotheses [2], [3]. The glutamate hypothesis focusing on N-methyl-D-aspartate (NMDA) glutamate receptor hypofunction has shown a number of promising leads [4]. NMDA receptor mediates glutamate-related cell signaling among neural cells. When the receptor is activated, transcription factors such as CREB (cAMP response element-binding) is mobilized to modulate long-term potentiation, long-term memory, synaptic plasticity and cell survival status. Based on this hypothesis, animal models treated by noncompetitive NMDA receptor antagonists, such as dizocilpine (MK-801) and phencyclidine, are widely used in schizophrenia research [5]. It has been shown that MK-801 treated rats demonstrate both positive and negative symptoms of schizophrenia [6].

Our previous proteomic study scrutinized cortical synaptosome proteins in subchronic MK-801 treated rats and revealed dysfunctions in energy metabolism in these rats [7]. Although alterations in brain energy metabolism have been found in human proteomic studies for schizophrenia [8], the exact metabolism pathways involved in the dysfunction have not been identified yet. This prompted us to further investigate metabolite levels in the same rat model to delineate the involved pathways which would provide insights to the pathology of schizophrenia. In the past, several studies concerning with certain metabolites have been conducted with the brain tissue extract of the MK-801 treated rats, finding that neurotransmitter metabolism in glial–neuronal interactions was impaired [9]–[12]. Metabolomics, as a modern systems biology approach, is different from the studies focusing on individual metabolites. It monitors entire pattern of low molecular weight compounds and models the global metabolic status of the samples. In the present study, we used proton magic angle spinning nuclear magnetic resonance (1H MAS NMR) spectroscopy to scan the overall metabolite signals in cortex and hippocampus of MK-801 treated rats. 1H MAS NMR spectroscopy has the advantage of a nondestructive procedure that can detect metabolites directly in the intact tissues. Cortex and hippocampus are two brain tissues that are rich of NMDA receptors and thus are responsive to MK-801, which helps us to identify the typical metabolism dysfunctions induced by MK-801. Multivariate statistics and ingenuity pathways analyses (IPA) were employed in data processing. The result was further combined with our previous proteomic data in IPA for a more systematic view on metabolomic observations.

## Materials and Methods

### Animal Model and Ethics Statement

All animal handling and procedures were performed in accordance with the Guide for the Care and Use of Laboratory Animals once the study received approval by the Institutional Animal Care and Use Committee at Shanghai Jiao Tong University Bio-X institutes, Shanghai, China. All surgery was performed aseptically and every attempt was made to minimize pain and discomfort.

23 male Sprague-Dawley rats (220–250 g) were randomly divided in two groups. Rats in the control group (n = 11) were injected subcutaneously with physiological saline 3.5 ml/kg (0.9% wt/vol NaCl [aqueous]) and those in the treatment group (n = 12) with 0.7 mg/kg MK-801 (Research Biochemicals, Natick, Massachusetts) (saline as vehicle) for 10 days. We chose the dose of 0.7 mg/kg as it produced the proper animal model [9] and it was the same dose as our previous work [7]. The volumes of MK-801 or saline were adjusted according to the body weight of each individual animal. The rats were kept in a 12∶12-hour light/dark cycle with food and water available ad libitum. On day 11, approximately 24 hours after the final injection, the rats were killed by cervical dislocation (http://www.ccac.ca/en_/standards/guidelines). The brains were quickly removed and the frontal and parietal lobe of cortex and hippocampus were excised from the brain and immediately snap-frozen in liquid nitrogen and stored at −80°C pending analysis. These operations were typically processed within 5–10 min to limit post-mortem changes in the metabolite content of the samples.

### 1H MAS NMR Spectroscopic Analysis

Each frozen 15–20 mg intact sample was rinsed with D_2_O solution (1 mg/ml) and then rapidly inserted into a zirconia 4 mm outer diameter rotor (Bruker Analytische GmbH, Rheinstetten, Germany). D_2_O provided a field-frequency lock. 1H MAS NMR data were recorded on a Bruker AVANCE spectrometer with a field strength of 500.13 MHz. Samples were spun at 5 KHz and maintained at 298 K throughout the experiment to minimize temperature-dependent metabolic changes [13]. In order to suppress broad signals from macromolecules, such as proteins, and hence to focus the subsequent analysis on the relatively small molecules, Carr–Purcell–Meiboom–Gill (CPMG) spin-echo pulse sequence [D–90°–(τ–180°–τ)n–FID, where FID is free induction decay] with a fixed spin–spin relaxation delay, 2 nτ of 64 ms (n = 128, τ = 400 µs), was applied to acquire 1H MAS NMR spectra of all samples. Typically, 256 transients were collected into 64 K data points with a spectral width of 30 ppm and an acquisition time of 2.18 s per scan. Prior to Fourier transformation, the FIDs were multiplied by an exponential weighting function corresponding to a line broadening factor of 1 Hz. Manual phase and baseline correction was performed using TOPSPIN software (Bruker Biospin GmbH, version 2.1). The spectra were referenced to lactate (CH3 δ = 1.325). Metabolites were identified with reference to the literatures [14]–[17] and the standard spectra database in HMDB (http://www.hmdb.ca).

### Data Reduction and Statistical Analyses

A bucket size of 0.01 ppm was chosen to reduce the spectra data using AMIX software (Bruker Biospin GmbH, version 3.8.6). The regions 0–0.6 ppm (no signal peaks), 1.1–1.23 ppm (ethanol), 3.62–3.7 ppm (ethanol), 4.54–5.0 ppm (water) and 8.3–20 ppm (no signal peaks), were excluded. The reduced spectral data were then normalized to a constant sum for each spectrum.

The univariate Student’s *t*-test was applied to each bin to evaluate its variation between groups. To account for multiple comparisons, the p-value from each *t*-test was mapped to a Storey-Tibshirani’s q-value using the “qvalue” package in R platform (http://www.r-project.org) to estimate the false discovery rate of the test when it’s called significant. The bins with q-values lower than 0.2 were regarded as significantly changed bins [18]. For multivariate statistical analysis, bucket tables were imported to SIMCA-P software (version 11.5; Umetrics, Umea, Sweden). In the software, the univariance scaling method was employed to avoid over-weighting of peaks from metabolites of high concentrations. Orthogonal Partial Least Squares-Discriminant Analysis (OPLS-DA) was conducted to separate MK-801 group from the control group, optimizing the discovery of treatment-related metabolites. For each PLS model, the explained variation (R^2^) and goodness of prediction (Q^2^) were given by the software for model evaluation. A cross validation was additionally conducted to test the predictability of the models. Firstly, a test set was constructed using three observations from each class. The left observations constituted the training set. A model was built with the training set and was used to predict the test set’s class membership with a cutoff of 1.5 (1 for the treated & 2 for the control). This was repeated for four times and the average percentage of correct classification was calculated.

To better interpret the results from OPLS-DA, back-scaled coefficient plots were drawn using R software (http://www.r-project.org/): firstly, the coefficients of the first OPLS component were back-transformed by multiplying all values by the respective variable standard deviation; secondly, the back-transformed coefficients were plotted and colored according to respective VIP (Variable Importance for the Projection) values generated by SIMCA-P software for the model. The scale range of the colors was set using the maximum and the minimum of the VIP values.

Variables (bins) that with a Student’s *t*-test q-value lower than 0.2 or an OPLS-DA VIP value higher than 1.5 were selected as the MK-801 treatment related bins. Those bins were assigned with corresponding metabolites. An average fold change of the bins from the same metabolite was calculated as the ratio of the average bin value in treated group to that in control group, indicating an overall change direction of the treatment related metabolite. This result was then imported in Ingenuity Pathways Analysis (IPA) software for molecular pathway and network analysis.

### Molecular Pathway and Network Analysis in IPA

Ingenuity Pathways Analysis (IPA; http://www.ingenuity.com) is a web-based software application that identifies biological pathways and functions relevant to bio-molecules of interest. To scrutinize the systematic influence of the treatment related metabolites, we uploaded the metabolite lists (with KEGG IDs) and the change directions of these metabolites onto an IPA server. Canonical pathways and molecular interaction networks were generated based on the knowledge sorted in the Ingenuity Pathway Knowledge Base. A ratio of the number of metabolites that map to the canonical pathway divided by the total number of molecules that map to the pathway was displayed. Fisher’s exact test was used to calculate a p-value determining the probability that the association between the metabolites and the canonical pathway was explained by chance alone. The network score was based on the hypergeiometric distribution and was calculated with the right-tailed Fisher’s Exact Test. The higher a score was, the more relevant the eligible submitted molecules were to the network. Integrated analysis of results from the present study and our previous proteomic study was also conducted in IPA by uploading a combined list of the treatment related metabolites and proteins onto the IPA server.

## Results

**Figure 1:**
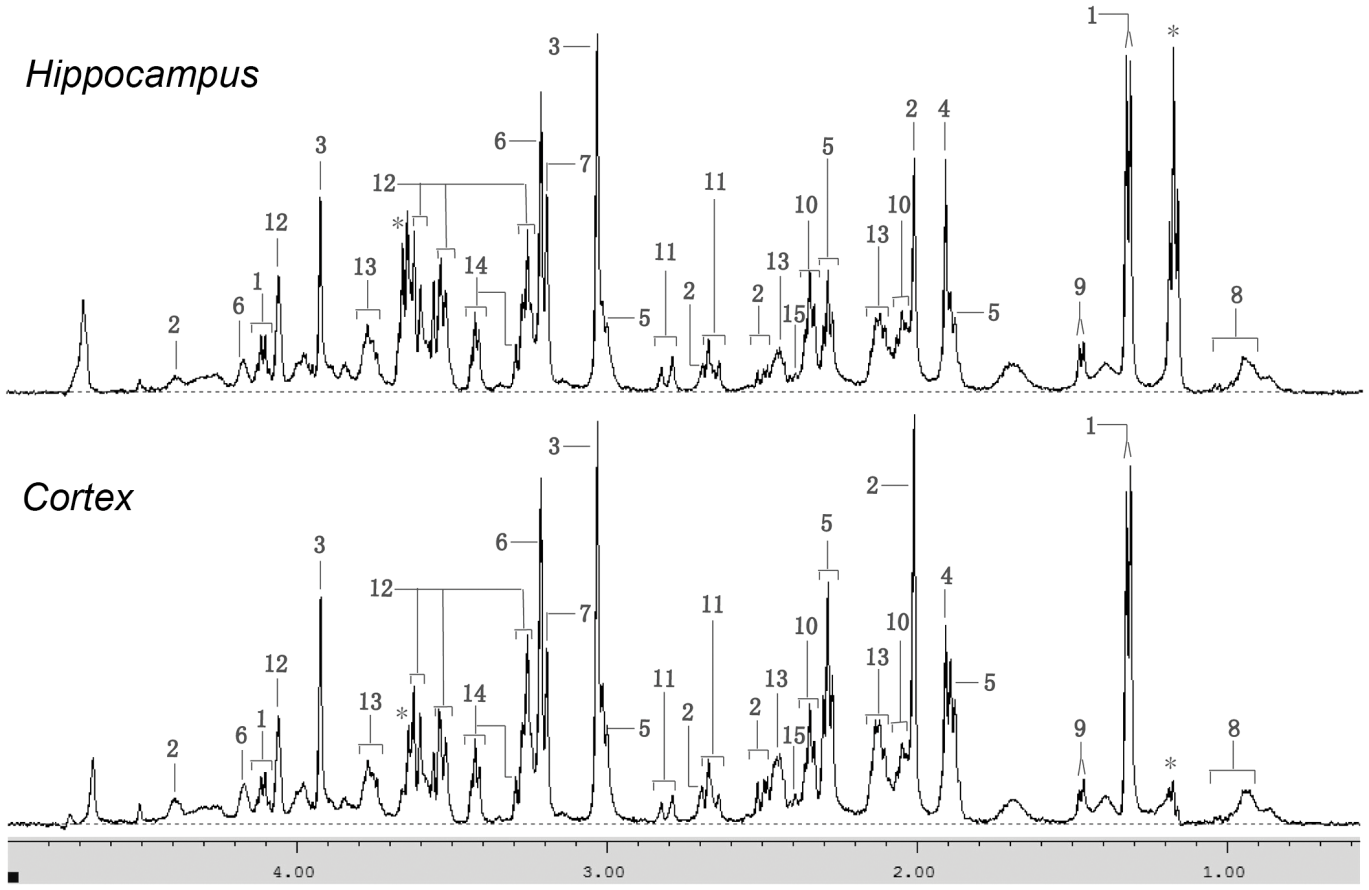
500 MHz CPMG 1H MAS NMR spectra (1–5 ppm). Top: hippocampus; bottom: rat cortex. Signals at 1.1–1.23 ppm & 3.62–3.7 ppm (labeled with “*”) correspond to ethanol, a contaminant from tool disinfection during sample preparation. These regions were absent from statistical analyses. Keys: 1, lactate; 2, N-acetylaspartate (NAA); 3, creatine; 4, acetate; 5,γ-Aminobutyric acid (GABA); 6, phophorylcholine (PC); 7, choline; 8, L-valine/L-leucine/L-isoleucine; 9, L-alanine; 10, L-glutamate; 11, L-aspartate; 12, myoinositol; 13, L-glutamine; 14, taurine; 15, succinate.

**Figure 2:**
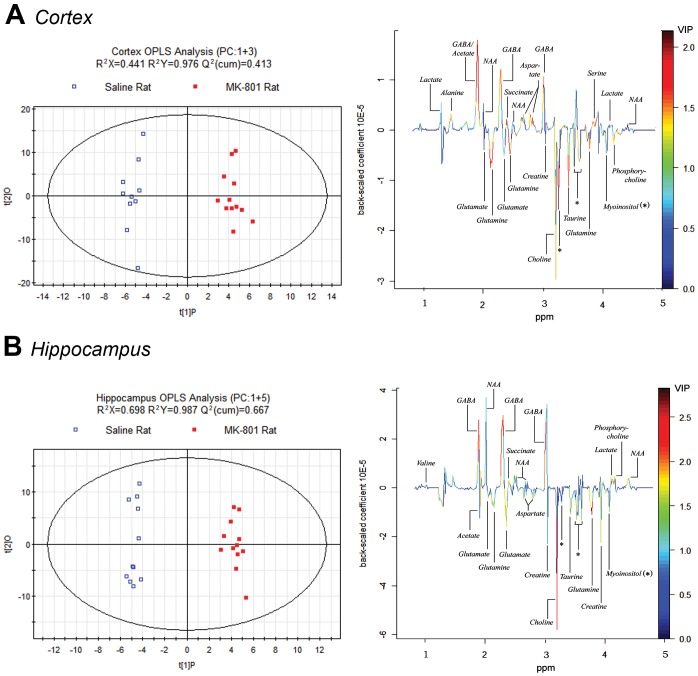
OPLS-DA score plots and back-scaled coefficient plots(1–5 ppm). Corresponding metabolites have been noted to the peaks in back-scaled loading plot of cortex (A) or hippocampus (B). The positive or negative phase of a peak represents increase or decline of the level of relevant metabolite. VIP values of the bins can be roughly judged from their colors: hot colored bins (e.g. bins of GABA in red) had high VIP values and contributed more than the cold colored ones (e.g. bins of myoinositol in blue) for the inter-group discrimination.

The 1H MAS NMR spectra from cortex were similar to that from hippocampus (Figure 1). After data reduction, 325 bins (variables) were obtained from the spectra. Bins from the MK-801 treated group and the control group were compared by Student’s *t*-test. 48 bins had p-values lower than 0.05 in the cortex, of which 44 had q-values lower than 0.2. 34 bins had p-values lower than 0.05 in the hippocampus, of which 11 had q-values lower than 0.2 (Table S1 & S2). Compared with bins in the hippocampus, more bins in the cortex showed statistically significant changes. Multivariate OPLS-DA analysis was implemented to directly search for treatment related metabolites and the results were displayed in the forms of score plots and back-scaled loadings plots (Figure 2). The score plots showed a clear separation between the MK-801 treated group and the control group in both cortex and hippocampus (with R^2^X = 0.441, Q^2^Y = 0.413 and R^2^X = 0.698, Q^2^Y = 0.677, respectively). Further validation showed that cortex models could predict class membership well with an accuracy of 83.3% and hippocampus models with an accuracy of 82.6%. In the cortex of the MK-801 treated rats, the back-scaled loading plot shows increased levels of lactate, acetate, L-alanine, L-aspartate, GABA, NAA, scyllitol, L-serine and succinate, and decreased levels of citrate, glutamine, glutamate, myoinositol, choline, phosphorylcholine, creatine and taurine. Similar result was seen in the hippocampus but with some differences, such as acetate and L-aspartate levels which were elevated in the cortex but decreased in the hippocampus. The VIP values of the bins can be roughly judged from the colors indicated in the back-scaled loading plots. Warm colored bins (e.g. bins of GABA in red) with high VIP value contributed more than the cold colored ones (e.g. bins of myoinositol in blue) in the inter-group discrimination.

We listed all the treatment related variables (bins) with either VIP value >1.5 or q-value <0.2 (Table S1 & S2). Those variables (bins) with higher VIP values in OPLS analysis tended to have lower p-values and q-values in the Student’s *t*-tests. Most of the bins could be assigned to corresponding metabolites. Table 1 is an extract of Table S1 & S2 that lists all these treatment related metabolites found in cortex and hippocampus of MK-801 treated rats. The change direction of a metabolite was indicated along with an average fold change value of the bins of the same metabolite. Among these metabolites, GABA, succinate and NAA were up-regulated in both the cortex and hippocampus; levels of myoinositol and glutamine were consistently decreased in the two brain tissues; concentrations of L-aspartate, phosphocholine (PC) and L-serine changed differently in the cortex and hippocampus, suggesting a variation in response to MK-801 in different brain areas.

**Table 1:** Treatment related metabolites in subchronic MK-801 treated rats’ cortex and hippocampus.

Cortex			Hippocampus		
Metabolites	ID in KEGG	Change Direction (FC)	Metabolites	ID in KEGG	Change Direction (FC)
Scyllo-inositol	C06153	▴ (1.15)	L-Valine	C00183	▴ (1.19)
Acetate/GABA	C00033/C00334	▴ (1.13)	NAA	C01042	▴ (1.16)
L-aspartate	C00049	▴ (1.13)	GABA	C00334	▴ (1.14)
NAA	C01042	▴ (1.13)	Phophorylcholine	C00588	▴ (1.14)
L-alanine	C00041	▴ (1.12)	Lactate	C00186	▴ (1.10)
GABA	C00334	▴ (1.09)	Succinate	C00042	▴ (1.09)
L-serine	C00065	▴ (1.07)	L-serine	C00065	▾ (0.94)
Succinate	C00042	▴ (1.07)	Creatine	C00300	▾ (0.92)
L-glutamine	C00064	▾ (0.92)	L-glutamine	C00064	▾ (0.91)
Myo-inositol	C00137	▾ (0.92)	L-glutamate	C00025	▾ (0.89)
Phophorylcholine	C00588	▾ (0.90)	Myo-inositol	C00137	▾ (0.89)
Taurine	C00245	▾ (0.88)	L-aspartate	C00049	▾ (0.82)
Citrate	C00158	▾ (0.84)	Choline	C00114	▾ (0.80)

**Note:** “▴” indicates increase and “▾” indicates decrease; GABA: γ-Aminobutyric acid; NAA: N-acetylaspartate. FC: the average fold change of the discriminant bins denoted as the same metabolite.

**Figure 3:**
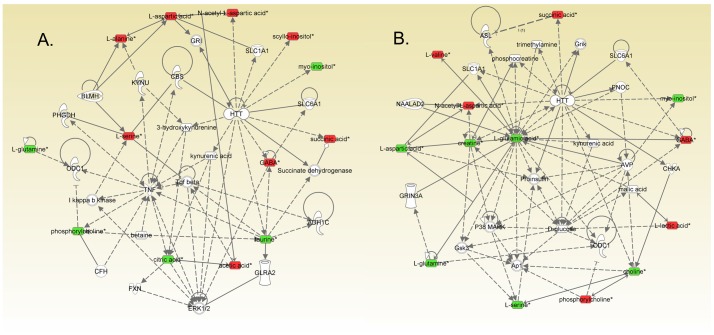
Networks associated with subchronic MK-801 treatment identified by Ingenuity Pathway Analysis. For clarity, proteins and metabolites are presented with different geometric shapes in the relevant network in cortex (A) or hippocampus (B). Metabolite symbols with red were up-regulated while green were down-regulated. Proteins and treatment unrelated metabolites are shown as clear. Dotted lines show indirect interactions or regulations between the two parties, while solid lines show direct physical interactions (such as binding) of the two parties.

IPA analysis was applied with treatment related metabolites to explore systematic influences of subchronic MK-801 treatment. The top ten altered pathways were generated and are listed in Table 2. The common pathways shared by the two brain regions were alanine and aspartate metabolism, glutamate metabolism, GABA receptor signaling, nitrogen metabolism and glycine, serine and threonine metabolism. In the network function analysis, treatment related metabolites in cortex and hippocampus tended to gather into one single network, respectively (Figure 3). The two networks were similar and shared the same functions, i.e., amino acid metabolism, molecular transport and small molecule biochemistry.

**Table 2:** Top 10 influenced ingenuity canonical pathways generated from IPA analyses.

Cortex			Hippocampus		
Top 10 Canonical Pathways	*P*-value	Ratio	Top 10 Canonical Pathways	*P*-value	Ratio
Alanine and Aspartate Metabolism	1.60E−06	5/88 (0.057)	GABA Receptor Signaling	6.75E−06	3/56 (0.054)
Glutamate Metabolism	9.05E−05	4/78 (0.051)	Aminoacyl-tRNA Biosynthesis	2.36E−05	5/84 (0.06)
Taurine and Hypotaurine Metabolism	1.85E−04	3/47 (0.064)	Glycine, Serine and Threonine Metabolism	2.65E−05	5/150 (0.033)
Aminoacyl-tRNA Biosynthesis	4.59E−04	4/84 (0.048)	Glutamate Metabolism	9.05E−05	4/78 (0.051)
Citrate Cycle	8.21E−04	3/58 (0.052)	Cyanoamino Acid Metabolism	5.12E−04	3/64 (0.047)
GABA Receptor Signaling	8.29E−04	2/56 (0.036)	Arginine and Proline Metabolism	1.13E−03	4/183 (0.022)
Nitrogen Metabolism	1.56E−03	3/133 (0.023)	D-glutamine and D-glutamate Metabolism	1.24E−03	2/27 (0.074)
Glycine, Serine and Threonine Metabolism	6.74E−03	3/150 (0.027)	Butanoate Metabolism	1.39E−03	3/132 (0.023)
Sulfur Metabolism	8.25E−03	2/61 (0.033)	Alanine and Aspartate Metabolism	1.39E−03	3/88 (0.034)
Selenoamino Acid Metabolism	1.06E−02	2/77 (0.026)	Nitrogen Metabolism	1.56E−03	3/133 (0.023)

**Note:** GABA: γ-Aminobutyric acid; the “Ratio” was calculated from dividing the number of metabolites that map to the canonical pathway by the total number of molecules that map to the pathway; the “*P*-value” was calculated from Fisher’s exact test. See the Materials and Methods section for details.

Our previous proteome study revealed 49 proteins altered in the cortical synaptosomes of subchronic MK-801 treated rats (Table S3). We combined those differentially expressed proteins with treatment related metabolites in the cortex of this study and carried out an additional IPA analysis [7]. The top network function was still the amino acid metabolism, molecular transport and small molecule biochemistry, while the top canonical pathway switched to the Krebs cycle (Table 3).

**Table 3:** Top 5 influenced canonical pathways and top 5 networks generated from IPA analysis combining previous proteomic data.

Top 5 Canonical Pathways			Top 5 Networks	
Name	*P*-value	Ratio	Associate Network Functions	Score
Citrate Cycle	3.47E−14	9/57 (0.158)	Amino Acid Metabolism, Molecular Transport, Small Molecule Biochemistry	43
Alanine and AspartateMetabolism	1.12E−09	7/82 (0.085)	Lipid Metabolism, Small Molecule Biochemistry, Nucleic Acid Metabolism	29
Oxidative Phosphorylation	3.03E−08	8/159 (0.05)	Cellular Assembly and Organization, Cell Cycle, Biliary Hyperplasia	22
Glutamate Metabolism	4.35E−08	6/75 (0.08)	Genetic Disorder, Metabolic Disease, Cardiovascular Disease	19
Mitochondrial Dysfunction	5.6E−07	7/175 (0.04)	Cell Cycle, Reproductive System Development and Function,Cellular Development	2

**Note:** the “Ratio” was calculated from dividing the number of metabolites that map to the canonical pathway by the total number of molecules that map to the pathway; the “*P*-value” was calculated from Fisher’s exact test; the “Score” indicates the association between the molecules and the network. See the Materials and Methods section for details.

## Discussion

In this study, we employed modern metabolomic method on the platform of 1H MAS NMR to scrutinize metabolite traits in cortex and hippocampus of subchronic MK-801 treated rats, a NMDA receptor hypofunction animal model for schizophrenia. We found that metabolites, not only neurotransmitters but also those involved in energy metabolism, were altered in this schizophrenia animal model.

### NMDA Receptor Hypofunction Causes Disturbance to Glutamate Homeostasis

Glutamate was reduced in the hippocampus and had a trend of decrease in the cortex in our study (Table 1 & Figure 2). The change of glutamate in the brain of schizophrenia patients has been the subject of discussion since 1980 but no consensus has so far been achieved [19]–[24]. Animal models offer valuable evidence in this field. Acute injection of MK-801 in rats has been shown to cause an elevation of glutamate in certain brain regions [25], [26]. However, in line with our result, a mouse model subject to 7-day subchronic MK-801 injection showed decreased extracellular glutamate level in the prefrontal cortex [27]. Similar results were obtained in most of brain subareas of MK-801 treated Sprague-Dawley rat model [10]. This implies a potential dynamic regulation of glutamate level in response to the length of MK-801 treatment, which is suggestive for human studies since a comprehensive down-regulation of glutamate synthesis was found in chronic schizophrenia patients [28].

### Glutamate Related Metabolic Pathway Involving Energy Metabolism was the Top Altered Pathways

**Figure 4:**
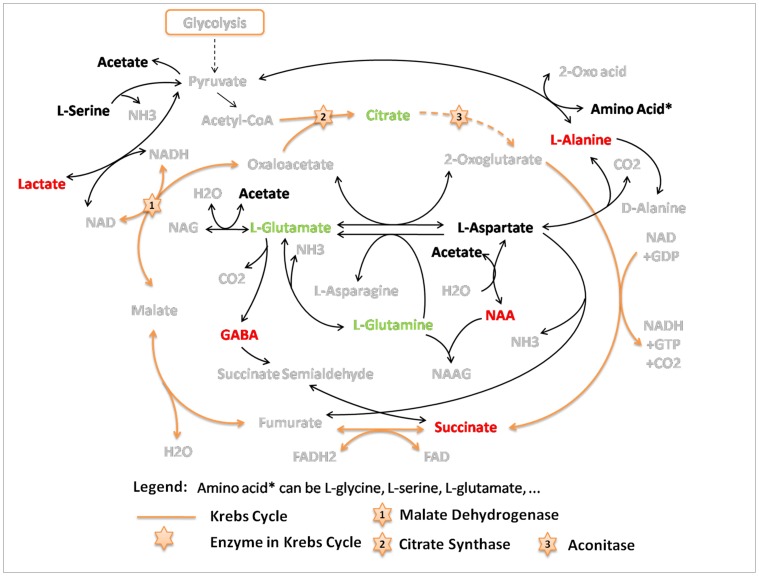
Top altered pathways in rat cortex and hippocampus induced by subchronic MK-801 treatment. Metabolite variation information here refers to the trends of changes (not only statistically significant changes). Metabolites in red (green) increased (decreased) in two brain areas; metabolites in black changed in opposite directions in two brain regions. Solid-lined arrows indicate direct biochemical reactions, while dashed-lined arrows represent a skip of intermediate reactions.

IPA analysis revealed 5 commonly altered pathways in the rat cortex and hippocampus following MK-801 treatment. Based on their biochemical relationships, we integrated the pathways into a brief plot that included most of the altered metabolites (Figure 4). This plot shows that glutamate and glutamine were concurrently down-regulated, but GABA was up-regulated in both brain areas. GABA is a typical inhibitory neurotransmitter and GABAergic neurons are sensitive to glutamate elevation [29]. Thus, an elevation of glutamate can stimulate GABAnergic neurons to release GABA which inversely inhibits glutamate synthesis. This kind of negative feedback might be involved in the dynamic regulation of glutamate in respond to MK-801 treatment as previously mentioned. Moreover, like the glutamate, GABA’s direction of disturbance in schizophrenia or related animal models is still unclear. Discrepancies were found among studies with different samples. For instance, similar to this study, elevated GABA level has been found in chronic schizophrenia patients [30] but no differences in the density of parvalbumin-immunoreactive(PV-ir) GABAergic neurons in cortex was seen in a postmortem study of schizophrenia [31]. Moreover, decreased GABA level was found in rat’s prefrontal cortex after 5-day repeated treatment of phencyclidine which is another NMDA receptor antagonist [32]. Repeated phencyclidine treatment also reduced density of PV-ir GABAergic neurons in rat’s hippocampus 6 weeks after the dosing [33]. Another postmortem study of schizophrenia has identified deficit of GABAergic neurons in frontal cortex [34]. To ravel these conflicts, more systematically designed studies are required to delineate the dysfunction of GABAergic neurons in schizophrenia.

Besides neurotransmitter dysregulation, we also observed alterations in the Krebs cycle: the succinate was elevated, while citrate was declined. Succinate is an important intermediate in the Krebs cycle, and can be formed from GABA through GABA shunt (Figure 4). GABA shunt is a characteristic pathway in GABAergic neurons. It allows GABA carbon skeleton to enter the Krebs cycle via succinate. The elevation of succinate is possibly associated with the excess of GABA. A number of enzymes in Krebs cycle have been found abnormal due to MK-801 treatment, such as citrate synthase [35], malate dehydrogenase and aconitase [36]. Our previous proteome study also found alterations in the Krebs cycle in the cortical synaptosome of subchronic MK-801 treated rats, pointing to a dysfunction of brain mitochondrial energy metabolism caused by MK-801 [7]. Lactate was increased in our study, which may result from a deficiency of energy supply.

Aspartate and alanine metabolism was also altered in the treated rats. L-aspartate was increased in the rat cortex but decreased in the hippocampus, while L-alanine was enriched in both brain areas. It has been reported that aspartate and alanine metabolism was disturbed in the dorsal prefrontal cortex of schizophrenia patients [37]. The elevation of L-alanine may play different roles in different types of neuron. Through transamination reactions, L-alanine can be converted into pyruvate and utilized as a metabolic fuel in GABAergic neurons [38]. Considering the enhanced GABA shunt and impaired Krebs cycle identified in our study, the elevation of alanine level might be required to meet the energy demand in activated GABAergic neurons. L-alanine is also regarded as a carrier of ammonia nitrogen from glutamatergic neurons to astrocytes using a flux of lactate in the opposite direction to account for the balance of C3 carbon skeleton [38], [39]. From this aspect, the increase of L-alanine along with lactate indicates a stirring transamination activity in glutamatergic neurons and glials, which has already been suggested above as a glutamate centered amino acid metabolism disturbance.

We found that levels of acetate and L-serine increased in cortex but decreased in hippocampus (Figure 4), suggesting that the response to MK-801 varied in different brain tissues. L-Serine is required for the synthesis of glycine and D-serine, both of which are NMDA receptor co-agonists. Potential roles of L-serine have been suggested in schizophrenia [40], [41]. Acetate is a common anion in biology and is mainly utilized in the form of acetyl coenzyme A for energy metabolism or acetylizations. Acetylization of L-aspartate turns out N-Acetylaspartate (NAA), an abundant metabolite in brain neurons. NAA was up-regulated in our study both in the cortex and hippocampus. Increased NAA was also found in the hippocampus of schizophrenia patients in a MRS study [22]. NAA was recently reported to be a major storage and transport form of acetyl coenzyme A in the nervous system [42], linking the accumulation of NAA to insufficient downstream utilization, i.e., the impaired Krebs cycle indentified in this study.

### Result in the Network Function Analysis of IPA

In the literature-based network analysis, i.e. IPA analysis, the main functions of our two networks constructed from the cortex and hippocampus respectively are of the same function, i.e., amino acid metabolism, molecular transport and small molecule biochemistry. The networks encompass many biomolecules important in schizophrenia research, such as kynurenic acid which is an endogenous glutamate antagonist [43]. HTT (huntingtin) is one of the core molecules appearing in both networks. It has also been featured in our previous proteomic study [7]. HTT is a primary protein involved in Huntington’s disease. Wild-type HTT protects neurons from NMDA receptor-mediated excitotoxicity [44], while polyglutamine-expanded HTT sensitizes NMDA receptors toward excitotoxicity [45] and hampers glial glutamate transport capacity [46]. Given the close interaction of HTT with the NMDA receptor mediated glutamate signaling system, its relationship to schizophrenia, though currently unclear, deserves future investigation.

### Metabolite-protein Integrated IPA Analysis

We did metabolite-protein integrated IPA analysis in order to expand our vision from the metabolic “dimension” to the protein-metabolic “space” and to reveal otherwise hidden implications using solely metabolomic data. The final top network function was the same as the metabolomic result while the top canonical pathway turned out to be the Krebs cycle. This result not only agrees well with our metabolomic conclusion that glutamate related and energy metabolism were the top altered pathways responding to subchronic MK-801 injection but also stresses the involvement of energy metabolism such as the Krebs cycle in the MK-801 induced dysfunctions. Xiao et.al came up with a similar result through a LC-MS based metabolomic study of the prefrontal cortex of subchronic phencyclidine treated rats [32]. Phencyclidine is another widely used NMDA receptor antagonist. Compared with our study, though different technical platforms for metabolite detecting and different algorithms for data mining were employed in Xiao’s study, they also found disturbances of metabolites such as GABA, glutamate and glutamine in the treated rats and concluded that subchronic phencyclidine treatment would induce compromised glutamatergic neurotransmission as well as disruption of metabolic pathways linked to glutamate in the rats model.

### Conclusion

Our study revealed a series of treatment related metabolites in the cortex and hippocampus of subchronic MK-801treated schizophrenia rat model. The disturbed pathway was a highly interconnected glutamate and energy metabolic pathway characterized by down-regulated glutamate synthesis and disturbed Krebs cycle. The disturbances on glutamate neurotransmitter system and energy metabolism are both well-recognized hypotheses of schizophrenia [47], [48]. This study reveals innate biochemical connections between those two theories. Compared to the glutamate hypothesis, energy metabolism dysfunction hypothesis is less discussed and deserves more attention in schizophrenia researches. It also suggested that future studies focusing on either hypothesis take the other one into consideration for a better understanding of the biochemical basis of schizophrenia etiology. In addition, this study proved that metabolomics, as a modern systems biology method, is an efficient and robust knowledge discovery approach for disease studies. Seeing that only cortex and hippocampus which are MK-801 high-binding regions were used here, studies concerning with other MK-801 low-binding brain areas, such as cerebellum and brainstem, are recommended for further validations and explorations.

## Supporting Information

Table S1 **Variables (indicated as bins) in rat cortex with either VIP value >1.5 or q-value <0.2.** Note: “▴” indicates increase and “▾” indicates decrease; figures in bold indicate VIP values >1.5 or q-values <0.2; p-value was generated from Student’s t-test; q-value indicates the false discovery rate when the particular Student’s t-test was called significant; VIP (Variable Importance for the Projection) value was generated from OPLS analysis; average fold change was computed by summing related bins of the metabolite and calculating the ratio of the average sum in treated group to that in control group.
(XLSX)

Table S2 **Variables (indicated as bins) in rat hippocampus with either VIP value >1.5 or q-value <0.2.** Note: “▴” indicates increase and “▾” indicates decrease; figures in bold indicate VIP values >1.5 or q-values <0.2; p-value was generated from Student’s t-test; q-value indicates the false discovery rate when the particular Student’s t-test was called significant; VIP (Variable Importance for the Projection) value was generated from OPLS analysis; average fold change was computed by summing related bins of the metabolite and calculating the ratio of the average sum in treated group to that in control group.
(XLSX)

Table S3 **Significantly changed proteins found in our previous proteomic study.**
(XLSX)

## References

[1] Knapp M, Mangalore R, Simon J (2004) The global costs of schizophrenia. Schizophr Bull 30: 279–293. 15279046 10.1093/oxfordjournals.schbul.a007078

[2] Rogers DP, Goldsmith CA (2009) Treatment of schizophrenia in the 21st Century: beyond the neurotransmitter hypothesis. Expert Rev Neurother 9: 47–54. 19102668 10.1586/14737175.9.1.47

[3] Vita A, Dieci M, Giobbio GM, Tenconi F, Invernizzi G (1997) Time course of cerebral ventricular enlargement in schizophrenia supports the hypothesis of its neurodevelopmental nature. Schizophr Res 23: 25–30. 9050125 10.1016/s0920-9964(96)00085-0

[4] Coyle JT (2006) Glutamate and schizophrenia: beyond the dopamine hypothesis. Cell Mol Neurobiol 26: 365–384. 16773445 10.1007/s10571-006-9062-8 PMC11881825

[5] Bubenikova-Valesova V, Horacek J, Vrajova M, Hoschl C (2008) Models of schizophrenia in humans and animals based on inhibition of NMDA receptors. Neurosci Biobehav Rev 32: 1014–1023. 18471877 10.1016/j.neubiorev.2008.03.012

[6] Rung JP, Carlsson A, Ryden Markinhuhta K, Carlsson ML (2005) (+)-MK-801 induced social withdrawal in rats; a model for negative symptoms of schizophrenia. Prog Neuropsychopharmacol Biol Psychiatry 29: 827–832. 15916843 10.1016/j.pnpbp.2005.03.004

[7] Zhou K, Yang Y, Gao L, He G, Li W, et al.. (2010) NMDA Receptor Hypofunction Induces Dysfunctions of Energy Metabolism And Semaphorin Signaling in Rats: A Synaptic Proteome Study. Schizophr Bull. 10.1093/schbul/sbq132 PMC3329985 21084551

[8] Martins-de-Souza D, Harris LW, Guest PC, Bahn S (2011) The role of energy metabolism dysfunction and oxidative stress in schizophrenia revealed by proteomics. Antioxid Redox Signal 15: 2067–2079. 20673161 10.1089/ars.2010.3459

[9] Eyjolfsson EM, Brenner E, Kondziella D, Sonnewald U (2006) Repeated injection of MK801: an animal model of schizophrenia? Neurochem Int 48: 541–546. 16517016 10.1016/j.neuint.2005.11.019

[10] Eyjolfsson EM, Nilsen LH, Kondziella D, Brenner E, Haberg A, et al.. (2010) Altered (13)C glucose metabolism in the cortico-striato-thalamo-cortical loop in the MK-801 rat model of schizophrenia. J Cereb Blood Flow Metab. 10.1038/jcbfm.2010.193 PMC3063632 21081956

[11] Kondziella D, Brenner E, Eyjolfsson EM, Markinhuhta KR, Carlsson ML, et al (2006) Glial-neuronal interactions are impaired in the schizophrenia model of repeated MK801 exposure. Neuropsychopharmacology 31: 1880–1887. 16395297 10.1038/sj.npp.1300993

[12] Kondziella D, Brenner E, Eyjolfsson EM, Sonnewald U (2007) How do glial-neuronal interactions fit into current neurotransmitter hypotheses of schizophrenia? Neurochem Int 50: 291–301. 17084946 10.1016/j.neuint.2006.09.006

[13] Tsang TM, Griffin JL, Haselden J, Fish C, Holmes E (2005) Metabolic characterization of distinct neuroanatomical regions in rats by magic angle spinning 1H nuclear magnetic resonance spectroscopy. Magn Reson Med 53: 1018–1024. 15844164 10.1002/mrm.20447

[14] Fan TWM (1996) Metabolite profiling by one- and two-dimensional NMR analysis of complex mixtures. Progress in Nuclear Magnetic Resonance Spectroscopy 28: 161–219.

[15] Pears MR, Cooper JD, Mitchison HM, Mortishire-Smith RJ, Pearce DA, et al (2005) High resolution 1H NMR-based metabolomics indicates a neurotransmitter cycling deficit in cerebral tissue from a mouse model of Batten disease. J Biol Chem 280: 42508–42514. 16239221 10.1074/jbc.M507380200

[16] Tsang TM, Haselden JN, Holmes E (2009) Metabonomic Characterization of the 3-Nitropropionic Acid Rat Model of Huntington’s Disease. Neurochemical Research: 1–11. 10.1007/s11064-008-9904-5 19148750

[17] Salek RM, Xia J, Innes A, Sweatman BC, Adalbert R, et al (2010) A metabolomic study of the CRND8 transgenic mouse model of Alzheimer’s disease. Neurochem Int 56: 937–947. 20398713 10.1016/j.neuint.2010.04.001

[18] Ting L, Cowley MJ, Hoon SL, Guilhaus M, Raftery MJ, et al (2009) Normalization and statistical analysis of quantitative proteomics data generated by metabolic labeling. Mol Cell Proteomics 8: 2227–2242. 19605365 10.1074/mcp.M800462-MCP200 PMC2758752

[19] Kim JS, Kornhuber HH, Schmid-Burgk W, Holzmuller B (1980) Low cerebrospinal fluid glutamate in schizophrenic patients and a new hypothesis on schizophrenia. Neurosci Lett 20: 379–382. 6108541 10.1016/0304-3940(80)90178-0

[20] van Elst LT, Valerius G, Buchert M, Thiel T, Rusch N, et al (2005) Increased prefrontal and hippocampal glutamate concentration in schizophrenia: evidence from a magnetic resonance spectroscopy study. Biol Psychiatry 58: 724–730. 16018980 10.1016/j.biopsych.2005.04.041

[21] Purdon SE, Valiakalayil A, Hanstock CC, Seres P, Tibbo P (2008) Elevated 3T proton MRS glutamate levels associated with poor Continuous Performance Test (CPT-0X) scores and genetic risk for schizophrenia. Schizophr Res 99: 218–224. 18248960 10.1016/j.schres.2007.11.028

[22] Lutkenhoff ES, van Erp TG, Thomas MA, Therman S, Manninen M, et al (2010) Proton MRS in twin pairs discordant for schizophrenia. Mol Psychiatry 15: 308–318. 18645571 10.1038/mp.2008.87

[23] Tayoshi S, Sumitani S, Taniguchi K, Shibuya-Tayoshi S, Numata S, et al (2009) Metabolite changes and gender differences in schizophrenia using 3-Tesla proton magnetic resonance spectroscopy (1H-MRS). Schizophr Res 108: 69–77. 19097753 10.1016/j.schres.2008.11.014

[24] Ohrmann P, Siegmund A, Suslow T, Spitzberg K, Kersting A, et al (2005) Evidence for glutamatergic neuronal dysfunction in the prefrontal cortex in chronic but not in first-episode patients with schizophrenia: a proton magnetic resonance spectroscopy study. Schizophr Res 73: 153–157. 15653258 10.1016/j.schres.2004.08.021

[25] Loscher W, Honack D, Fassbender CP (1991) Regional alterations in brain amino acids after administration of the N-methyl-D-aspartate receptor antagonists MK-801 and CGP 39551 in rats. Neurosci Lett 124: 115–118. 1677457 10.1016/0304-3940(91)90835-h

[26] Brenner E, Kondziella D, Haberg A, Sonnewald U (2005) Impaired glutamine metabolism in NMDA receptor hypofunction induced by MK801. J Neurochem 94: 1594–1603. 16045441 10.1111/j.1471-4159.2005.03311.x

[27] Zuo DY, Zhang YH, Cao Y, Wu CF, Tanaka M, et al (2006) Effect of acute and chronic MK-801 administration on extracellular glutamate and ascorbic acid release in the prefrontal cortex of freely moving mice on line with open-field behavior. Life Sci 78: 2172–2178. 16280137 10.1016/j.lfs.2005.09.022

[28] Theberge J, Al-Semaan Y, Williamson PC, Menon RS, Neufeld RW, et al (2003) Glutamate and glutamine in the anterior cingulate and thalamus of medicated patients with chronic schizophrenia and healthy comparison subjects measured with 4.0-T proton MRS. Am J Psychiatry 160: 2231–2233. 14638596 10.1176/appi.ajp.160.12.2231

[29] Gonzalez-Burgos G, Lewis DA (2008) GABA neurons and the mechanisms of network oscillations: implications for understanding cortical dysfunction in schizophrenia. Schizophr Bull 34: 944–961. 18586694 10.1093/schbul/sbn070 PMC2518635

[30] Ongur D, Prescot AP, McCarthy J, Cohen BM, Renshaw PF (2010) Elevated gamma-aminobutyric acid levels in chronic schizophrenia. Biol Psychiatry 68: 667–670. 20598290 10.1016/j.biopsych.2010.05.016 PMC2942977

[31] Cotter D, Landau S, Beasley C, Stevenson R, Chana G, et al (2002) The density and spatial distribution of GABAergic neurons, labelled using calcium binding proteins, in the anterior cingulate cortex in major depressive disorder, bipolar disorder, and schizophrenia. Biol Psychiatry 51: 377–386. 11904132 10.1016/s0006-3223(01)01243-4

[32] Xiao X, Dawson N, Macintyre L, Morris BJ, Pratt JA, et al (2011) Exploring metabolic pathway disruption in the subchronic phencyclidine model of schizophrenia with the Generalized Singular Value Decomposition. BMC Syst Biol 5: 72. 21575198 10.1186/1752-0509-5-72 PMC3239845

[33] Jenkins TA, Harte MK, Reynolds GP (2010) Effect of subchronic phencyclidine administration on sucrose preference and hippocampal parvalbumin immunoreactivity in the rat. Neurosci Lett 471: 144–147. 20097262 10.1016/j.neulet.2010.01.028

[34] Reynolds GP, Beasley CL (2001) GABAergic neuronal subtypes in the human frontal cortex–development and deficits in schizophrenia. J Chem Neuroanat 22: 95–100. 11470557 10.1016/s0891-0618(01)00113-2

[35] Meloni BP, Van Dyk D, Cole R, Knuckey NW (2005) Proteome analysis of cortical neuronal cultures following cycloheximide, heat stress and MK801 preconditioning. Proteomics 5: 4743–4753. 16252307 10.1002/pmic.200500107

[36] Paulson L, Martin P, Nilsson CL, Ljung E, Westman-Brinkmalm A, et al (2004) Comparative proteome analysis of thalamus in MK-801-treated rats. Proteomics 4: 819–825. 14997502 10.1002/pmic.200300622

[37] Middleton FA, Mirnics K, Pierri JN, Lewis DA, Levitt P (2002) Gene expression profiling reveals alterations of specific metabolic pathways in schizophrenia. J Neurosci 22: 2718–2729. 11923437 10.1523/JNEUROSCI.22-07-02718.2002 PMC6758309

[38] Schousboe A, Sonnewald U, Waagepetersen HS (2003) Differential roles of alanine in GABAergic and glutamatergic neurons. Neurochem Int 43: 311–315. 12742074 10.1016/s0197-0186(03)00017-2

[39] Waagepetersen HS, Sonnewald U, Larsson OM, Schousboe A (2000) A possible role of alanine for ammonia transfer between astrocytes and glutamatergic neurons. J Neurochem 75: 471–479. 10899921 10.1046/j.1471-4159.2000.0750471.x

[40] Ozeki Y, Pickard BS, Kano S, Malloy MP, Zeledon M, et al (2011) A novel balanced chromosomal translocation found in subjects with schizophrenia and schizotypal personality disorder: altered l-serine level associated with disruption of PSAT1 gene expression. Neurosci Res 69: 154–160. 20955740 10.1016/j.neures.2010.10.003 PMC3049551

[41] Ohnuma T, Sakai Y, Maeshima H, Hatano T, Hanzawa R, et al (2008) Changes in plasma glycine, L-serine, and D-serine levels in patients with schizophrenia as their clinical symptoms improve: results from the Juntendo University Schizophrenia Projects (JUSP). Prog Neuropsychopharmacol Biol Psychiatry 32: 1905–1912. 18835577 10.1016/j.pnpbp.2008.07.022

[42] Ariyannur PS, Moffett JR, Manickam P, Pattabiraman N, Arun P, et al (2010) Methamphetamine-induced neuronal protein NAT8L is the NAA biosynthetic enzyme: implications for specialized acetyl coenzyme A metabolism in the CNS. Brain Res 1335: 1–13. 20385109 10.1016/j.brainres.2010.04.008

[43] Erhardt S, Schwieler L, Nilsson L, Linderholm K, Engberg G (2007) The kynurenic acid hypothesis of schizophrenia. Physiol Behav 92: 203–209. 17573079 10.1016/j.physbeh.2007.05.025

[44] Leavitt BR, van Raamsdonk JM, Shehadeh J, Fernandes H, Murphy Z, et al (2006) Wild-type huntingtin protects neurons from excitotoxicity. J Neurochem 96: 1121–1129. 16417581 10.1111/j.1471-4159.2005.03605.x

[45] Sun Y, Savanenin A, Reddy PH, Liu YF (2001) Polyglutamine-expanded huntingtin promotes sensitization of N-methyl-D-aspartate receptors via post-synaptic density 95. J Biol Chem 276: 24713–24718. 11319238 10.1074/jbc.M103501200

[46] Faideau M, Kim J, Cormier K, Gilmore R, Welch M, et al (2010) In vivo expression of polyglutamine-expanded huntingtin by mouse striatal astrocytes impairs glutamate transport: a correlation with Huntington’s disease subjects. Hum Mol Genet 19: 3053–3067. 20494921 10.1093/hmg/ddq212 PMC2901144

[47] Martins-de-Souza D, Harris LW, Guest PC, Bahn S (2011) The role of energy metabolism dysfunction and oxidative stress in schizophrenia revealed by proteomics. Antioxid Redox Signal 15: 2067–2079. 20673161 10.1089/ars.2010.3459

[48] Moghaddam B, Javitt D (2012) From revolution to evolution: the glutamate hypothesis of schizophrenia and its implication for treatment. Neuropsychopharmacology 37: 4–15. 21956446 10.1038/npp.2011.181 PMC3238069

